# Editorial: Diversity and equity in autism research

**DOI:** 10.3389/fpsyt.2026.1974136

**Published:** 2026-09-22

**Authors:** Kristina Lopez, Ana D. Dueñas, Rachel K. Schuck

**Affiliations:** 1 Arizona State University School of Social Work, Phoenix, AZ, United States; 2 University of Washington College of Education, Seattle, WA, United States; 3 Stanford University Department of Psychiatry and Behavioral Sciences, Stanford, CA, United States

**Keywords:** autism, diversity, equity, inclusion, intersectionality

## Introduction

Autism research increasingly shapes who is diagnosed, and how individuals are treated and supported; yet the field has largely overlooked the diversity of the people it studies. Methodology and rigor have advanced, but diversity and equity remain scarce or narrowly applied. Race, ethnicity, gender, sexual orientation, class, religion, culture, type of disability, and their intersections shape diagnosis, intervention, and support, yet are often absent from analyses (1, 2). Consequences are well documented: racial/ethnic minorities, females, and low-resourced autistic children are diagnosed later (3, 4) and face substantial unmet needs (5, 6). Closing these gaps requires researchers to not only ensure diverse populations are included in their studies but to also design research that confronts equity head-on. In this Research Topic eleven articles address various aspects of diversity and equity in autism research.

Three papers lay the conceptual groundwork, each examining a different mechanism producing inequity: exclusion from research participation, the erasure of intersecting identities within broad demographic categories, and ideological assumptions embedded in “evidence-based” practice. Together, they shift the conversation from documenting disparities to explaining the systemic processes that generate them, setting the stage for the studies that follow. Passarini et al. reframe compliance in clinical and research settings as an equity issue. Their narrative review of Augmentative and Alternative Communication (AAC) shows communication barriers primarily drive exclusion, with an estimated 90% of children with neurodevelopmental conditions absent from research protocols despite available supportive tools. The authors model a concrete way to reduce “underrepresentation bias”: building accommodation into research design, rather than treating complex-communication populations as too difficult to include. This extends the call’s interest in community-inclusive studies to research infrastructure itself. Luelmo and Castellón make visible a population usually subsumed under broader ethnic categories. Studying Indigenous Mixtec/Zapotec-speaking caregivers in a California school district, they show autism identification disparities aren’t fully explained by a Latine/Hispanic-versus-White binary: Mixtec-speaking caregivers were markedly less likely than Spanish-speaking non-Indigenous caregivers to obtain a diagnosis before their child’s IEP. Qualitative data trace this to compounding exclusions– lack of Mixtec interpretation, unfamiliarity with special education, institutional dependence–masked when Indigenous families are aggregated into pan-ethnic labels. This paper operationalizes intersectionality directly, showing within-group heterogeneity can conceal disparities that between-group comparisons miss. Castellón et al. supply theoretical scaffolding for these mechanisms. Their perspective piece traces how discredited claims linking bilingualism to cognitive deficit became embedded in autism intervention practice, persisting because ableism and racism co-construct a deficit lens around multilingual autistic children. They identify the same structural problem Passarini et al. raise from a different angle: systematic exclusion of non-English-speaking participants from intervention research has produced an evidence base reinforcing English-only, assimilationist practice.

Three papers explore areas that can impact quality of life among diverse groups of autistic individuals and their families. Two of these papers focus specifically on mental health; one looks at employment. Zheng and Liu surveyed caregivers of autistic children in China and found that feelings of alienation were related to depression amongst participants, but that family resilience – particularly family communication/cohesion, social support, and faith/spirituality – moderated this relationship such that greater resilience attenuated the relationship. Also using a survey methodology, Sivathasan et al. explored the relationships between sexual minority status and gender identity and variables associated with mental health conditions amongst autistic adults. Gender minorities reported more anxiety and depression symptoms than cisgender participants, and both gender and sexual minorities were more likely to have anxiety and depression diagnoses than cisgender-heterosexual autistic individuals, controlling for other demographic variables. Lastly, Kan et al. interviewed autistic adults in Singapore about their workplace needs, with an emphasis on how large language models (LLMs) might provide opportunities for support. Reflexive thematic analysis generated two themes that emphasized the prevalence of neurotypical assumptions in work processes and social interactions, as well as the need for inclusive, person-centered, systemic change. Participants thought LLMs might be helpful tools but stressed that they needed to be informed by autistic individuals. These studies highlight the complex interplay between individual characteristics, social interactions, and societal expectations in autistic individuals and their loved ones. They also emphasize the need to specifically study these relationships in diverse groups of the autism community instead of assuming that findings with one group will generalize to another.

Lastly, four articles included in this Research Topic outline culturally adapted interventions for diverse and underserved families of children with autism. Venegas and Magaña describe a culturally adapted parent-mediated intervention for low-resourced and ethnically/racially diverse parents and young children with autism. The intervention, *ASD Screening and Parent Engagement* (ASPEN), integrated time-constraints among families in the study and highlighted the importance of cultural adaptations to meet the needs of an underserved sample of families. Zhu et al. present a telepractice intervention of *Prevent, Teach, and Reinforce for Families* (PTR-F) for Chinese American families of young autistic children in the U.S. The culturally adapted PTR-F was conducted with six Chinese American families and found to be accepted by and beneficial for families to address their children’s challenging behaviors. The use of telepractice also took into account the time limitations families experienced while continuing to deliver culturally adapted autism intervention. Xu et al. studied the pre, post, and follow-up time points of a culturally informed intervention program for underserved Chinese immigrant families raising children with autism. The virtual delivery of the culturally informed program allowed researchers to expand the scope of Chinese immigrant families that could be reached. Finally, Williams et al. describes the development and iterative, user-centered pilot testing of *Asking for Help* (A4H), an app designed to increase mental health literacy for Black Autistic Youth. Adaptations based on participant feedback – such as the autistic youth wanting simplified, affirming language – are described, and future studies to assess efficacy are outlined. By culturally adapting interventions and delivering them through flexible and novel methods, these three studies exemplify the positive impact of intentional inclusion of diversity and equity in autism research featured in this Research Topic.

## Conclusion

Together, these eleven articles demonstrate that centering diversity and equity strengthens autism research methodologically and ethically, ultimately improving diagnosis, intervention, and support for all autistic individuals and their families.

## References

[1] Steinbrenner JR McIntyre N Rentschler LF Pearson JN Luelmo P Jaramillo ME . Patterns in reporting and participant inclusion related to race and ethnicity in autism intervention literature: Data from a large-scale systematic review of evidence-based practices. Autism. (2022) 26:2026–40. doi: 10.1177/13623613211072593 35068190 PMC9596958

[2] Robertson RE Sobeck EE Wynkoop K Schwartz R . Participant diversity in special education research: Parent-implemented behavior interventions for children with autism. Remedial Special Educ. (2017) 38:259–71. doi: 10.1177/0741932516685407

[3] Pham AV Charles LC . Racial disparities in autism diagnosis, assessment, and intervention among minoritized youth: Sociocultural issues, factors, and context. Curr Psychiatry Rep. (2023) 25:201–2011. doi: 10.1007/s11920-023-01417-9 37004631

[4] Hodge MA Sutherland R Boulton KA Baracz SJ Ong N Bennett B . Focusing on autism symptoms masks sex-specific needs of autistic children: An example from the Sydney Child Neurodevelopment Research Registry. Autism. (2025) 29:1318–32. doi: 10.1177/13623613241303550 39704418

[5] Papoudi D Jørgensen CR Guldberg K Meadan H . Perceptions, experiences, and needs of parents of culturally and linguistically diverse children with autism: A scoping review. Rev J Autism Dev Disord. (2021) 8:195–212. doi: 10.1007/s40489-020-00210-130311153

[6] Koffer Miller KH Cooper DS Song W Shea LL . Self-reported service needs and barriers reported by autistic adults: Differences by gender identity. Res Autism Spectr Disord. (2022) 92:1–8. doi: 10.1016/j.rasd.2022.10191642574925

